# A large community outbreak of gastroenteritis associated with consumption of drinking water contaminated by river water, Belgium, 2010

**DOI:** 10.1017/S0950268814001629

**Published:** 2014-07-25

**Authors:** T. BRAEYE, K. DE SCHRIJVER, E. WOLLANTS, M. van RANST, J. VERHAEGEN

**Affiliations:** 1 Epidemiology of Infectious Diseases, Scientific Institute of Public Health, Brussels, Belgium; 2 Department of Infectious Disease Control, Agency of Care and Health, Antwerp, Belgium; 3 Department of Epidemiology and Social Medicine, University of Antwerp, Antwerp, Belgium; 4 Laboratory of Clinical Virology, Rega Institute for Medical Research, Catholic University of Leuven, Leuven, Belgium; 5 Department of Microbiology, Catholic University of Leuven, Leuven, Belgium

**Keywords:** Gastroenteritis, outbreak, waterborne

## Abstract

On 6 December 2010 a fire in Hemiksem, Belgium, was extinguished by the fire brigade with both river water and tap water. Local physicians were asked to report all cases of gastroenteritis. We conducted a retrospective cohort study among 1000 randomly selected households. We performed a statistical and geospatial analysis. Human stool samples, tap water and river water were tested for pathogens. Of the 1185 persons living in the 528 responding households, 222 (18·7%) reported symptoms of gastroenteritis during the time period 6–13 December. Drinking tap water was significantly associated with an increased risk for gastroenteritis (relative risk 3·67, 95% confidence interval 2·86–4·70) as was place of residence. *Campylobacter* sp. (2/56), norovirus GI and GII (11/56), rotavirus (1/56) and *Giardia lamblia* (3/56) were detected in stool samples. Tap water samples tested positive for faecal indicator bacteria and protozoa. The results support the hypothesis that a point-source contamination of the tap water with river water was the cause of the multi-pathogen waterborne outbreak.

## INTRODUCTION

Tap water contaminated by gastrointestinal pathogens remains an important cause of gastrointestinal disease. In the European Union 86 enteric disease outbreaks associated with public drinking water supplies were reported from 1990 to 2004 [1]. In the USA 780 outbreaks were associated with drinking water from 1971 to 2006 [2]. Outbreaks are hard to detect and the number of cases associated with an outbreak varies. Waterborne disease is not limited to outbreaks. Sporadic cases probably represent a greater proportion of waterborne disease than cases related to outbreaks [1].

At the beginning of the 20th century, bacteria were most often identified as the cause of waterborne outbreaks. Nowadays viral and protozoal pathogens are commonly reported. Worldwide, 199 parasitic protozoa outbreaks with waterborne transmission were publicized during 2004–2010 [3]. Protozoa are also responsible for endemic disease associated with tap water [3–5]. A characteristic of pathogens associated with waterborne disease is a low infectious dose [6]. The bacteria most often found in North America and Europe in recent waterborne outbreaks are *Campylobacter* sp. and *Escherichia coli* [7–9]. In 2005 the most predominant parasitic protozoa isolated in waterborne outbreaks were *Cryptosporidium*, 60·3%, and *Giardia lamblia*, 35·2% [10]. Finally caliciviruses and viruses such as group A rotavirus have also been detected during waterborne outbreaks [11]. New methods for detecting norovirus (NoV) have resulted in increased detection of these pathogens [12–14].

Waterborne outbreaks have multiple causes, e.g. problems within the water system [15–17]. This can be failure of the disinfection system [11, 18], cracks in the service reservoir [19] or the mains [9], inappropriate connections between sewage- and drinking-water pipelines [20] or a pressure fall in the distribution system [8]. However, causes are not limited to problems with or failure of the system, e.g. the length of pipe run from the treatment works to the home, is correlated with the risk of disease [1]. Studies have described seasonal trends, with a higher proportion of waterborne outbreaks during spring and autumn, and associated these with (heavy) weather and agricultural activities [5, 21]. Often surface water is contaminated with runoff from regions with cattle and sheep [18].

On 6 December 2010 there was a fire in a textile factory in the centre of Hemiksem. Firefighters used water from two hydrants, connected to the tap water network, and from a unit ‘hydrosub’, which is used for pumping surface and river water. Car pumps collected water from both sources in a pressurized water tank. The hydrosub was connected to the river Vliet, a small river that flows to the river Scheldt. On 7 December three out of four routinely taken tap water samples in Hemiksem and Schelle suggested faecal contamination. Local general practitioners (GPs) reported an increase in consultations for gastroenteritis on 8 December. On 9 December the residents were advised not to consume or use tap water. The water avoidance notice was lifted on 20 December 2010. Tap water in the neighbouring municipalities of Hemiksem and Schelle (*n* = 18 620 residents), is supplied by one water supply company that uses purified ground water. The company takes care of collecting, cleaning and distributing drinking water. The surveillance, done according to the specific legislation, is a responsibility of the water company. Belgium has no specific surveillance system on waterborne outbreaks. We conducted an epidemiological study to describe the size and identify the source of the outbreak.

## METHODS

The study population comprised of all the residents of Hemiksem and Schelle. We included no other municipalities given that elsewhere no faecal contamination in routinely taken samples of drinking water had been reported, and no increase of gastroenteritis had been registered. Considering that symptoms of gastroenteritis can be very mild, we combined a physicians' case-finding survey with a randomly sampled survey among the subscribers of the water company in Hemiksem and Schelle.

### Case reporting by physicians

On 9 December all 18 local GPs were asked to report cases of gastroenteritis. Additionally, the emergency department of a neighbouring hospital was contacted and asked to report any resident of Hemiksem or Schelle who presented with gastroenteritis. Inclusion criteria were patients who lived in Hemiksem or Schelle, and who had symptoms of diarrhoea (⩾3 loose stools per 24 h) or vomiting from 6 to 13 December 2010.

### Retrospective cohort study

A retrospective cohort study was conducted among a randomly selected sample of 1000 households from Hemiksem and Schelle. The sample was selected from a list of customers of the water supply company. A household was defined as all persons living at the same address. Through a postal survey every household member was asked for his personal usage of tap water (consumption, cooking, teeth brushing, cleaning), the usage of other water (ground water, bottled water), symptoms, with a focus on gastrointestinal symptoms, and the onset date of these symptoms and treatments. Any person reporting diarrhoea (⩾3 loose stools per 24 h) and/or vomiting between 6 and 13 December 2010, was defined as a case. Patients with an onset of diarrhoea or vomiting between 14 and 31 December 2010 were considered as late or secondary cases. Non-cases were people who did not report any symptoms. Households in which symptoms of gastroenteritis were reported prior to 6 December 2010 and persons who spent time abroad in the week prior to the outbreak were excluded from the study. We did not correct for a baseline number of gastroenteritis cases as no adequate incidence data for gastroenteritis was present for this region and time period. Based on the address of the respondent, the shortest distance, in metres, to the site of the fire was calculated.

Descriptive statistics were calculated for the retrospective cohort study. We performed univariate analysis (cross tables, *χ*^2^ tests, linear trend analysis, relative risk) and multivariate analysis (Poisson regression). *P* < 0·05 was considered statistically significant. Variables associated with the outcome at *P* < 0·20 in univariate analysis were introduced in the multivariate regression model. The final multivariate model was built using backwards elimination [22]. The data were entered using EpiData v. 3.1 (EpiData Association, Denmark) and analysed using SAS v. 9.2 (SAS Institute Inc., USA) and R 2.14 (R Foundation, Austria). An additional analysis focusing on the geographical distribution of the cases was performed using the R package ‘sparr’ [23]. With this software the spatial density of the cases and the spatial density of non-cases was compared.

### Microbiological study

Patients included in the physicians' survey were contacted and asked to provide a stool sample. These stool samples were tested for pathogenic gastrointestinal bacteria on culture media for isolation of enteropathogens (MacConkey agar, XLD and CIN agar) in a laboratory with accreditation to ISO 15 189. Antigen tests were used to test for *Cryptosporidium* and *G. lamblia* (Xpect Immunochromatographic Assays, Oxoid, UK). For the detection of NoV two methods were used. Three stool samples were send to the Scientific Institute of Public Health, Brussels, where they used a real-time RT–PCR for the detection and differentiation of the two most important human genogroups of NoV, GI and GII. This method is described in ISO/TS 15 216–1: 2013. The other 53 stool samples were send to the Clinical Virology Laboratory, Leuven and were analysed with a NoV RT–PCR. Two pairs of specific primers G1SKF/G1SKR and COG2F/G2SKR, that amplify the capsid gene, were used to respectively detect NoV GI (330 bp) and NoV GII (387 bp) [24]. An in-house RT–PCR was developed for detection of astrovirus. The primer set used to amplify 272 bp in the capsid protein gene was HASTV-F: ACAGAAGAGCAACTCCATCGC and HASTV-R: TGACACCYTGTTTCCTGAGTTG. A RT–PCR was used for rotavirus and adenovirus detection as described previously [25, 26]. The analyses of the tap water were performed by the water supply company. Samples were tested for bacterial indicators of faecal contamination: non-specific coliforms, *Escherichia coli, Clostridium* sp. and *Pseudomonas aeruginosa*. In two river water samples protozoa were isolated by the department of Parasitology of the University of Ghent.

## RESULTS

### Physicians' case-finding study

All 18 GPs participated in the study. We included 603 patients; 326 (54%) were men and 277 (46%) women. The age ranged from 1 to 91 years with a median age of 36 years. Three hundred and ninety-seven (66%) cases lived in Hemiksem, and 206 (34%) in Schelle. Consultations for gastroenteritis peaked on 8 December 2010. Six patients were hospitalized. One patient, aged 91 years, died due to intestinal bleeding on 12 December after being admitted to the hospital for gastroenteritis on 9 December 2010.

### Retrospective cohort study

The questionnaire was distributed on 20 December. The response rate was 52·8% (*n* = 528). The responses included information on a total of 1185 household members. This is 6·6% of the total population (*n* = 18 620) of Hemiksem and Schelle (December 2010).

The respondents' age ranged from 0 to 99 years and the median age was 39 years. Gender was balanced with 48·1% males and 51·9% females. Out of all respondents, 37% regularly drank tap water, with an average of 3·7 glasses a day, 99% used tap water to brush their teeth, and 98% used tap water to wash vegetables and fruit. Gastrointestinal symptoms were reported by 36·5% (*n* = 432). The mean duration of illness was 4·1 days. Diarrhoea was the most frequently reported symptom, present in 65·4% of those reporting symptoms, fever in 15%, vomiting in 39% and nausea in 60%. Thirty-four respondents reported symptoms prior to 6 December.

A total of 222 [18·7%, 95% confidence interval (CI) 16·4–20·9] persons met the case definition (onset of symptoms 6–13 December) and 176 were late or secondary cases (onset after 13 December).

**Fig. 1.**
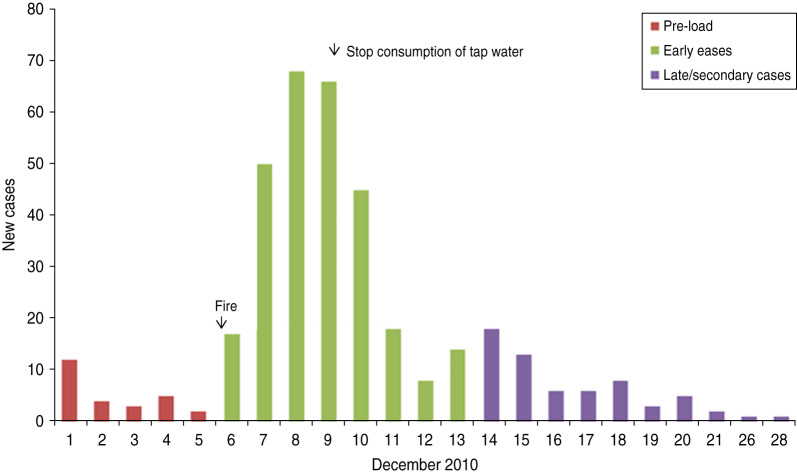
Cases over time, subscribers' study, Hemiksem and Schelle, 2010.

The incidence of gastroenteritis followed a steep incline from 6 to 8 December and peaked on 8 December (Fig. 1).

**Fig. 2.**
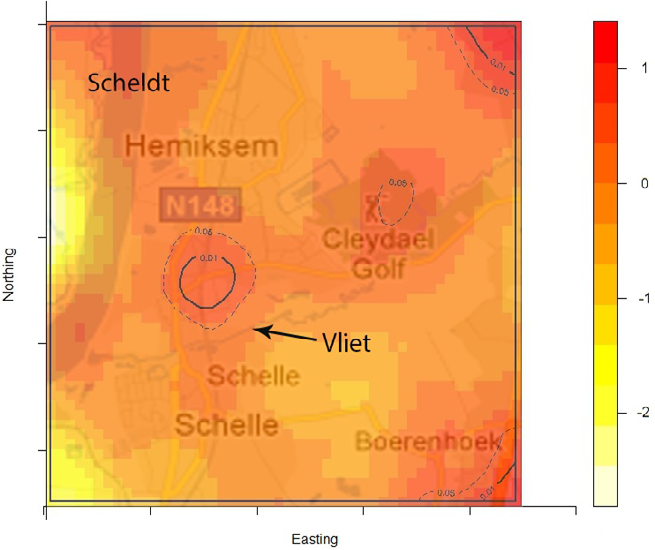
Density cases over non-cases. A redder region indicates a higher proportion of cases over non-cases; dotted line = contour line for significance *P* < 0·05; full line = contour line for significance *P* < 0·01; Hemiksem and Schelle, 2010.

**Fig. 3.**
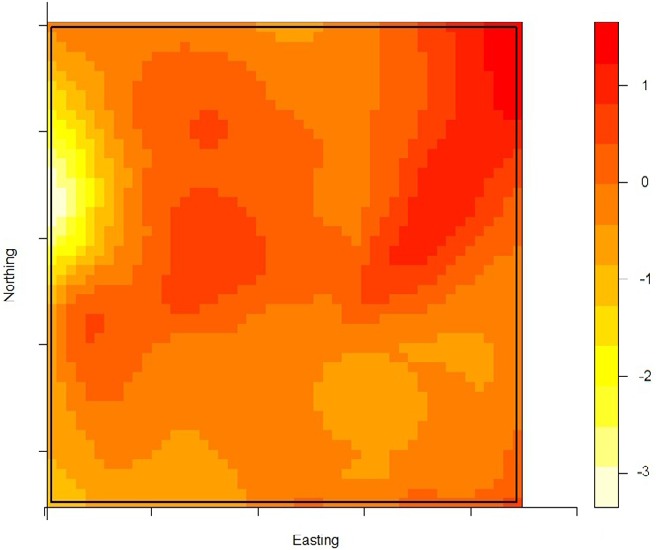
Density of tap-water drinkers over non-tap-water drinkers. A redder region indicates a higher proportion of tap-water drinkers; Hemiksen and Schelle, 2010.

The density of the cases (*n* = 222) over non-cases (*n* = 753) shows the highest proportion of cases slightly north of the site of the fire (Fig. 2). The density of self-reported ‘tap-water’ drinkers is compared to the density of ‘non-tap-water’ drinkers (see Fig. 3). A higher proportion of tap-water drinkers was observed east of Hemiksem.

### Univariate analysis

Drinking tap water was associated with gastrointestinal symptoms [relative risk (RR) 2·28, 95% CI 1·94–2·67] (Table 1). The relative risk was higher for cases (RR 3·67, 95% CI 2·86–4·70) than for secondary or late cases (RR 1·91, 95% CI 1·47–2·49) (Table 1). The distance from the home address of the respondent to the site of the fire was significantly shorter for cases compared to non-cases. Other variables such as gender, household size, age and the presence of young children (<12 years) in the family were not significant (Table 2).

**Table 1.** Contingency table for drinking tap water and the different case definitions (for 46 respondents information on tap water consumption was missing), Hemiskem and Schelle, 2010

		Non-cases	Secondary and late cases	Cases	Total
Tapwater					
No	*N*	549	95	74	718
	%	73·69	55·23	33·03	
Yes	*N*	196	77	148	421
	%	26·31	44·77	66·97	
Total	*N*	745	172	222	1139

**Table 2.** Univariate analysis of risk factors in cases compared to non-cases, Hemiksem and Schelle, 2010

Variable	RR	95% CI	*P* value
Tap water	3·67	2·86–4·70	0·001
Gender (male/female)	0·8	0·6–1·1	0·08
Distance to fire site (unit: 750 m)	0·89	0·82–0·97	0·04
Young children (<12 years)	0·9	0·7–1·2	0·6
Household size	Linear trend	–	0·43
Age	Linear trend	–	0·52

RR, Relative risk; CI, confidence interval.

### Multivariate analysis

The independent variables included in the multivariate model were: ‘glasses of tap water a day’, ‘location’ and ‘gender’. Location (distance from the site of the fire) as well as drinking tap water (glasses per day) were significantly (*P* < 0·05) associated with outcome in a Poisson regression model. For each glass of tap water consumed the risk was augmented by 21% (RR 1·21, 95% CI 1·16–1·26). For each 250 m that families lived further away from the site of the fire the risk diminished by 8·4% (RR 0·92, 95% CI 0·84–0·99) (Table 3).

**Table 3.** Multivariate analysis (Poisson regression) for cases compared to non-cases, Hemiksem and Schelle, 2010

Variable	RR	95% CI	*P* value
Tap water (glasses/day)	1·21	1·16–1·27	<0·001
Distance to fire site (unit: 250 m)	0·92	0·84–0·99	<0·04

RR, Relative risk; CI, confidence interval.

### Microbiological patient data

Sixteen of 56 stool samples were diagnosed with a pathogen. In the stool sample of 14 patients a single pathogen was detected: *Campylobacter* sp. was detected in one, NoV GI in two and NoV GII in seven patients. Rotavirus was detected in one patient and *G. lamblia* was detected in three samples. Two patients were diagnosed with a multi-pathogen infection: one patient with a double infection: NoV GI and GII, and one patient with a mixed infection with *Campylobacter jejuni*, NoV GI and GII.

### Environmental study

Between 8 and 25 December 2010, 625 water samples were analysed in Hemiksem and Schelle. In Hemiksem, high densities of faecal pollution indicators were detected in tap water samples: >200 c.f.u./100 ml for *E. coli* and >1100 c.f.u./100 ml for enterococci. Parasites, *G. lamblia* and *Cryptosporidium* sp. were isolated from samples taken from the river.

### Control measures

There were different authorities involved in controlling the outbreak and informing the population; the local municipal authorities, the environmental health team and the drinking water company. Bottled potable water was distributed to the population. Sanitation of water pipes (flushing, disinfection) was performed from 9 December 2010 to 25 December 2010.

Our research pointed towards an incident during the firefighting on 6 December 2010 as the cause of the contamination. Additional research eliminated other possible reasons by checking installations in the surrounding area. An investigation into the actions taken by firefighters pointed towards the absence of reflux valves as the most likely cause. Once this hazard was identified, a warning was sent to other fire brigades in Belgium to avoid similar incidents.

## DISCUSSION

We have described a large community outbreak of gastroenteritis associated with the consumption of water contaminated by river water during fire extinguishing. This is the first large waterborne outbreak described in Belgium. Waterborne disease, especially cryptosporidiosis, has been reported as a ‘work-related disease exposure for firefighters’, but it is the first time a community outbreak has been described in which the probable cause was associated with firefighting [27].

Our study estimated an attack rate of gastrointestinal infections of 18·7% without correction for baseline illness. The attack rate in our study can be explained by the high doses of pathogens in the tap water, a high daily consumption of tap water, a period of 3 days between the contamination and the water avoidance notice, and the low infectious dose of some pathogens. Attack rates in comparable studies vary and can be quite high, e.g. 51·4% in a tourist resort and new housing estate, 53% in a Finnish town [15, 20, 21, 28]. The number of cases per outbreak has been associated to the pathogens involved. *Giardia-* and *Cryptosporidium-*associated outbreaks have the lowest mean number of cases per outbreak (116 and 177, respectively). Viral outbreaks and outbreaks associated with *Campylobacter* sp. have the highest mean number of cases per outbreak (1545 and 1802, respectively) [1]. In a multi-pathogen outbreak, as we have described, one can expect an even higher number of cases. The number of hospitalizations was limited, but gastrointestinal disease can be associated with severe illness, especially in hig- risk patients [6] such as AIDS patients [29].

We found an unadjusted relative risk of 3·67 and the dose–response relationship was significant. A threefold increased risk for illness with the consumption of tap water is commonly associated with waterborne outbreaks [13, 16, 18, 19]. In *E. coli* and *Campylobacter* sp. outbreaks with relative risks of 11 were reported [7, 9].

In waterborne outbreaks, the strength of evidence implicating water as the cause of an outbreak is determined on the basis of findings from epidemiological and microbiological investigations. Tillett *et al*. developed a system of levels of evidence to link an outbreak to water [30]. We complement this by adding an important spatial component.

We observed a steep increase in reported gastroenteritis among the survey's responders on the 6 December which indicates a sudden contamination. Microbiological investigations during this increase identified multiple pathogens in the stool samples of patients. This is consistent with the hypothesis of a waterborne outbreak rather than a community-wide person-to-person transmission of e.g. a NoV [31]. Furthermore, tap water was a likely mode of transmission as there was a significant association between gastroenteritis and the consumption of tap water. Enterobacteriaceae were found in the tap water. We linked the firefighting to the outbreak by spatial and temporal analysis. We documented the association between place of residence and the risk of gastroenteritis by comparing the ratio of cases (*n* = 222) over non-cases (*n* = 753) with the ratio of tap-water drinkers over non-tap-water drinkers. We observed that one peak in the ratio of cases over controls was not accompanied by a high ratio of tap-water drinkers over non-tap-water drinkers. This cluster was located slightly north of the site of the fire. We have no data on quantitative difference of pathogen load in the pipelines.

A major weakness of this study is the incomplete microbiological investigation. Drinking water contaminated by sewage is known to result in mixed bacterial and viral infections and severe acute gastroenteritis regardless of the aetiological agents [17, 32]. Investigation of the tap water was limited to indicator bacteria for faecal contamination and a one-time detection of *Cryptosporidium* sp. and *G. lamblia.* Patients were not tested for protozoa. Detection of e.g. levels of serum antibody to *G. lamblia* or testing of patients for *Cryptosporidium* should be performed in this kind of outbreak investigation [33, 34]. No attempt was made to match the clinical and environmental pathogens. Pathogens from the environmental samples were not stored and therefore not available for further testing.

Since the incubation period of several gastrointestinal pathogens can vary from hours to weeks, we took the entire month of December 2010 as study period. However, the difference between cases and late or secondary cases is artificial and only used as a means to better analyse the data. Long incubation periods and secondary infections by person-to-person transmission are hard to differentiate.

Self-reported information on symptoms and water consumption is known to be biased, especially after media attention [35]. However, the media did not report on possible sources and the connection to the fire. The media only started to report on the outbreak after the water avoidance notice. We tried to collect objective information by contacting healthcare personnel. The questionnaire was sent rather late, on 20 December 2010, which could generate recall bias. Moreover, persons affected by the outbreak might be more inclined to respond to the survey leading to selection bias. No information was collected on the costs of this outbreak. Previous research shows that the costs for preventive measures clearly are smaller than the costs of a waterborne outbreak [36].

We did not investigate compliance with the water avoidance notice. A study in The Netherlands estimated compliance with a boil water advice at 81·8% [37].

Several improvements can be recommended such as the development of a surveillance system and a wider and more thorough microbiological investigation. Syndromic surveillance combined with water incident and consumer complaints data can be used for the timely detection of outbreaks, but this needs to be further evaluated [38]. More intense microbiological investigations are necessary both during standard controls and outbreaks. Previous studies have indicated that water-treatment technologies have become inadequate, and that a negative coliform test result does not guarantee that water is free from all pathogens, especially from protozoan agents [6]. Both enteric viruses, such as caliciviruses, and some protozoan agents, such as *Cryptosporidium*, are candidates for endemic transmission and outbreaks with these pathogens will go undetected with standard controls [39]. Furthermore, a more thorough microbiological investigation can be used to predict the likelihood of various transmission routes or vehicles [40].

This outbreak also highlights the need to rapidly connect an outbreak to its cause to reduce attack rates by implementing the correct measurements. This outbreak could have been detected quicker if additional surveillance systems had been in place. Rapid detection and intervention necessitates the collaboration between physicians, public health services, microbiologists and water providers.
